# Factors Explaining Disability-free Life Expectancy in Japan: the Proportion of Older Workers, Self-reported Health Status, and the Number of Public Health Nurses

**DOI:** 10.2188/jea.15.219

**Published:** 2005-11-07

**Authors:** Naoki Kondo, Takashi Mizutani, Junko Minai, Mari Kazama, Hisashi Imai, Yasuhisa Takeda, Zentaro Yamagata

**Affiliations:** 1 Department of Health Sciences, Interdisciplinary Graduate School of Medicine and Engineering, University of Yamanashi.

**Keywords:** Life Expectancy, Aged, Public Health Nursing, Socioeconomic Factors, self-rated health

## Abstract

BACKGROUND: Disability-free life expectancy (DFLE) data for 47 prefectures in Japan were reported in 1999; however, few studies have identified the factors associated with the length of the DFLE. The objective of this study was to elucidate the primary factors that explain differences in DFLEs in Japan.

METHODS: In our ecological study, 47 prefectures in Japan were used as units of analysis. The DFLEs for men and women at 65 years of age (DFLE65), calculated by Hashimoto et al using Sullivan’s method, were set as dependent variables. From various national surveys, 181 factors associated with demographics, socioeconomic status, health status and health behaviors, medical environment, social relationships, climate, and other areas were gathered as independent variables. Pearson’s or Spearman’s correlation coefficients were calculated to screen independent variables potentially associated with the DFLE65s. Then, multivariate linear regression analyses were conducted for the selected 24 independent variables after adjusting for the proportion of older people (65 years or more) and population density.

RESULTS: Multivariate linear regression analyses revealed that the large number of public health nurses per 100,000 population, a good self-reported health status, and a high proportion of older workers were significantly associated with long DFLE65s for both genders.

CONCLUSIONS: These three factors could potentially explain the differences in DFLE of the older population in Japan.

Summary measures for health expectancies such as disability-free life expectancy (DFLE) and disability-adjusted life expectancy (DALE) are widely used to evaluate and compare regional or country health statuses. The use of these indicators is recommended by the World Health Organization (WHO).^1^ In particular, DFLE measured by Sullivan’s method^1^ with dichotomous-weighted disability estimate is currently used by many countries;^3^^-^^5^ it is simpler to calculate by this method as compared to DALE (weighted by polychotomous disability levels^6^^,^^7^) and other health expectancies, such as multistate life table methods^8^^-^^12^ based on prospective data. The WHO reported in World Health Reports that among its member states, Japan is constantly ranked in the first tier of DALE.^13^ Hashimoto et al. measured DFLE in 47 prefectures in Japan in 1999.^14^^,^^15^ Their study results are used as the prefectural baseline data for work pertaining to “Healthy Japan 21” (the Japanese national health promotion activity advocated by the Ministry of Health, Labour and Welfare in 2000).^16^ However, further research that elucidates the factors associated with DFLE is required to facilitate the introduction of evidence-based health policies.

To date, two similar ecological studies have been conducted in Europe. A study in the United Kingdom revealed that social class, unemployment rate, and the percentage of non-white population were the main variables associated with the differences in DFLE at birth.^17^ Spanish data revealed that a higher illiteracy rate and higher percentage of smokers were associated with shorter DFLE at 65 years of age (DFLE65).^18^ In addition, certain other studies employing individual data indicated that people with low educational level have low DFLE.^6^^,^^19^^,^^20^ However, to our knowledge, few such studies have been conducted in Japan.

The objective of this study was to formulate hypotheses with respect to the primary factors that explain the DFLE differences found in Japan by using aggregated data from national surveys assessing various characteristics representative of Japan.

## METHODS

### Study settings

We carried out an ecological study in which 47 prefectures in Japan were used as units of analysis. The DFLE65 of men and women, which was calculated by Hashimoto et al.,^14^^,^^15^ were set as dependent variables. Hashimoto et al. have described the detailed procedure of estimation of the DFLE65; in brief, the DFLE indicators were calculated using Sullivan’s method. Disability was determined dichotomously (present or not) based on four national surveys conducted in 1995 and 1996. People with the following status were recognized as having disability: (1) patients who lived at home and needed help for any one of the following tasks: washing the face, brushing teeth, changing clothes, taking a meal, excreting, taking a bath, and walking (verified by the Comprehensive Survey of the Living Conditions of People on Health and Welfare^21^); (2) patients who needed to visit hospitals or clinics and required care similar to that mentioned above (verified by the Patient Survey^22^); (3) patients admitted to health service facilities for the aged (facilities for the elderly who do not require hospital treatment but need rehabilitation or nursing care under medical management) or a special elderly nursing home (facilities mainly for the bedridden elderly), verified by the Survey of Health Services Facilities for the Aged^23^ and the Survey of Social Welfare Facilities.^24^ Further, DFLE was calculated using a life table with indirect estimation using the ratio of stationary population in each age group stratified by sex and stationary population without disability in the same stratum.

### Selection of independent variables

We selected 181 variables obtained from multiple data sources, which were published by government organizations and public institutions between 1995 and 2001.^21^^,^^25^^-^^48^ Every conceivable variable of interest was considered. The 181 variables were categorized into seven groups: demographics, socioeconomic statuses (SES), health status and health behaviors, medical environment, social relationships, climate, and others.

### Statistical analysis

First, for descriptive observations and screening purposes, distributions of the selected variables were studied using histograms. Log-transformation was performed for the skewed variables of population density and unemployment rate. Then, correlation coefficients between DFLE65s of men and women and independent variables were calculated. For the correlation analyses, Pearson’s product moment correlation coefficient or Spearman’s rank correlation coefficient were used for the independent variables of normal or non-normal distribution, respectively. An independent variable with one absolute value of the correlation coefficient of 0.20 or more was selected as a factor that was potentially associated with the DFLE65s. The variables that were insignificant or irrelevant with regard to DFLE65 were excluded (e.g., the percentage of paved roads or electric energy consumption). Further, the linearity of each association with independent variables and DFLE65s were checked by analyzing scatter charts, and its heteroscedasticity was assessed by observing a residual plot. Finally, multivariate linear regression analyses were performed using the 24 selected variables. The proportion of older people (aged 65 years or more) and the population density (log-transformed) were included in the regression models as potential confounding factors. These data were included because it could theoretically affect other explanatory variables although the correlation between the two indicators and DFLE65s was not significantly strong. In fact, many explanatory variables were associated with them (e.g., Pearson’s correlation coefficients of the number of public health nurses (PHNs) per 100,000 population were -0.81 for log-transformed population density and 0.80 for the proportion of older people).

In Pearson’s correlation analysis and regression analysis, *Aomori* prefecture data was excluded as an outlier because its DFLE65s for both men (14.0 years) and women (17.3 years) were lower than that of the average of 47 prefectures (mean±standard deviation [SD] = 15.2±0.4 in men and 18.5±0.4 in women) by three SDs. In addition, residual analyses showed that the inclusion of Aomori prefecture data in the regression analysis deteriorated the linearity and equality of variance of the regression between DFLE65s and explanatory variables.

Self-reported health status was determined based on the question, “What do you think of your current health status?” in a Comprehensive Survey of the Living Conditions of People on Health and Welfare.^21^ The following five alternatives were provided as answers: good, relatively good, normal, relatively bad, and bad. In our study, the proportion of good self-reported health that was obtained by combining the proportion of “good” and “relatively good” self-reported health was used for analysis.

All the variables used contained no missing data. All *p* values are two tailed. All data analyses were performed using the SAS^®^ version 8.2 statistical package.

## RESULTS

Tables 1 and 2 show the descriptive statistics and Pearson’s or Spearman’s correlation coefficients between DFLE65s and independent variables for which the values of correlation coefficients were 0.20 or more. As shown in Table 1, the male and female DFLE65s were strongly correlated with each other. The log-transformed population density was inversely correlated with the DFLE65s, although the correlations were not statistically significant.

**Table 1.** Descriptive statistics and correlation coefficients between disability-free life expectancy at 65 years of age (DFLE65) and independent variables of demographics, socioeconomic status, and health status and health behaviors: the variables of correlation coefficients ≥ 0.20.

Variables	Units	Mean	SD	Minimum	Maximum	Correlation coefficients			Reference No.

						DFLE65 Men	DFLE65 Women	Methods^†^	
Demographics									
DFLE65 of men	years	15.2	0.4	14.0	16.0	1.00	0.89*	P	14
DFLE65 of women	years	18.5	0.4	17.3	19.4	0.89*	1.00	P	14
Age 65+ population^§^	%	16.3	2.8	10.1	21.7	0.15	0.17	P	45, 48
Population density (log-transformed)	per km^2^	5.8	1.0	4.3	8.6	-0.20	-0.18	P	45, 48
Average number of people^||^	per household	3.5	0.2	3.1	3.9	0.20	0.15	P	21
Age 65+ people living with their children	% of age 65+ population	18.3	10.6	2.0	44.1	0.20	0.15	S	21

Socioeconomic status									
Age 15+ workers	% of population	58.0	2.9	50.0	63.6	0.24	0.16	S	21
Age 65+ workers	% of population	23.9	3.4	15.8	31.3	0.42*	0.37*	P	21
Unemployment rate (log-transformed)	%	1.5	0.2	1.1	2.2	-0.24	-0.18	P	46
Ordinary income/expenditure balance ratio		86.6	5.6	75.1	102.9	-0.27^+^	-0.25^+^	P	27, 48
Regional difference of the consumer price index: Total	Tokyo = 100	93.1	2.3	88.5	100	-0.25^+^	-0.18	P	28, 48
Regional difference of the consumer price index: Food	Tokyo = 100	94.8	2.2	90.6	100	-0.18	-0.20	P	28, 48

Health status and health behaviors									
Self-reported health status: good	%	44.6	2.5	38.9	53.0	0.29^+^	0.26^+^	P	21
Average length of stay in hospital	days per patient	34.2	6.1	24.4	52.4	0.21	-0.16	S	33, 48
Have habitual exercises	% of age 20+ population	88.8	1.2	86.3	91.7	0.20	0.17	S	21
Female smokers	%	9.2	3.9	3.0	19.8	—	-0.22	S	35, 34

Pearson’s correlation coefficients were calculated for data without *Aomori* prefecture, which was identified as an outlier because of its extremely low DFLE65s.

SD: Standard deviation

+ : p<0.1

* : p<0.05

† : P: Pearson’s correlation coefficients; S: Spearman’s correlation coefficients

§ : Stated for reference despite its correlation coefficient < 0.20

|| : Except live-alone households

**Table 2.** Descriptive statistics and correlation coefficients between disability-free life expectancy at 65 years of age (DFLE65) and independent variables of medical environment, social relationships, climate, and others: the variables of correlation coefficients ≥ 0.20.

Variables	Units	Mean	SD	Minimum	Maximum	Correlation coefficients			Reference No.

						DFLE65 Men	DFLE65 Women	Methods^†^	
Medical environment									
Public health nurses (actually engaged in public health services)	per 100,000 population	35.4	9.8	15.9	57.9	0.45*	0.44*	P	47, 48
Hospitals (except psychiatric hospital and tuberculosis sanatorium)	per 100 km^2^ of habitation area	8.8	8.3	1.9	44.7	-0.10	-0.22	S	33, 48
Full-time physicians in hospital	per 100 beds	9.2	1.3	6.8	13.5	0.23	0.16	S	32, 48

Social relationships									
Social expenses	yen, per month	1462	328	854	2123	0.20	0.19	S	30, 48
Expenditures related to funerals	yen, per month	1325	897	152	3704	0.34*	0.19	S	30, 48

Agree that neighborly ties are strong in your community	%	55.6	5.9	40.3	67.2	0.23	0.25^+^	P	38

Have worries but no idea whom to consult	%	1.7	0.2	1.3	2.2	-0.35*	-0.24^+^	P	21

Climate									
Average difference of maximum-minimum temperature in a day	°C	8.3	1.4	5.5	13.7	0.27^+^	0.13	S	29

Others									
Number of traffic accident deaths	per 100,000 population	8.2	2.2	3.0	13.1	0.21	0.18	P	39, 48
Clearance rate of criminal law violators	%	25.0	8.2	11.5	46.1	0.18	0.20	S	40, 48
Cars	per 10,000 population	66.5	9.6	38.1	81.8	0.27^+^	0.18	S	42, 48
Private cars	per 10,000 population	45.6	6.4	25.7	57.6	0.27^+^	0.16	S	42, 48

Pearson’s correlation coefficients were calculated for data without Aomori prefecture, which was identified as an outlier because of its extremely low DFLE65s.

SD: Standard deviation

+ : p<0.1

*: p<0.05

† : P: Pearson’s correlation coefficients; S: Spearman’s correlation coefficients

In SES variables, the proportion of older workers was strongly correlated with DFLE65s of men and women (Pearson’s correlation coefficients = 0.42 and 0.37, respectively). Ordinary income/expenditure (I/E) balance ratio was negatively correlated with DFLE65s, while per capita income, industrial structure, and education level (the proportion of people whose academic backgrounds were high school or more and advancement rate to college) were not correlated with DFLE65s.

The proportion of good self-reported health and of those having exercising habitually were positively correlated with DFLE65s, in contrast with the average length of stay in hospitals (days per hospitalized patient) and the proportion of smokers in women, which were inversely correlated with them.

While examining the factors related to medical environment (Table 2), the number of PHNs per 100,000 population who were actually engaged in public health services was strongly correlated with DFLE65s (Pearson’s correlation coefficients = 0.45 for males, and 0.44 for females). The number of full-time physicians in hospital per 100 beds was also positively correlated with DFLE65s, whereas the numbers of clinics, beds, ambulances, and nurses (per unit of population and per unit of area) were not correlated with them.

With regard to social relationships, social expenses (costs for personal or social interactions such as the cost for gifts or banquets) and the proportion of people who agree that “neighborly ties are strong in your community” were positively correlated with DFLE65s; on the other hand, the proportion of people who “have some worries but have no idea whom to consult” was inversely correlated with DFLE65s.

The average difference in maximum-minimum temperatures in a day was positively correlated with DFLE65s, whereas other climatic indicators such as average temperature, sunshine duration, humidity, and rainfall level were not correlated. In other factors, the large number of cars per unit population was associated with high DFLE65 of men.

Table 3 shows the result of the multivariate linear regression analysis. It lists only the statistically significant (*p* < 0.05) variables. The following three variables were positively associated with both the male and female DFLE65 evenly adjusted for the potential confounders; i.e., the number of PHNs per 100,000 population, the proportion of older workers, and the proportion of good self-reported health. In addition, full-time physicians in hospital per 100 beds was positively associated with DFLE65 only in the case of men. The Pearson’s correlation coefficients of the number of PHNs and full-time physicians in hospitals with per capita income were -0.55 (*p* < 0.0001) and 0.64 (*p* < 0.0001), respectively, and those of the two variables with ordinary I/E balance ratio were -0.61 (*p* < 0.0001) and 0.19 (*p* = 0.20), respectively. Concerning the number of PHNs and physicians, additional multivariate models that included adjustments for these economic indicators were developed and demonstrated a significantly positive association between the number of PHNs and DFLE65s for both genders; however, the model nullified the association of the number of full-time physicians in hospital for DFLE65 for men.

**Table 3.** Result of multivariate linear regression analysis: explanatory variables significantly (p < 0.05) associated with disability-free life expectancy at 65 years of age for men and women.

	*β*	SE	Standardized *β*	p	Adjusted R^2^
Men					
Public health nurses (per 100,000 population)	0.035	0.008	1.11	< 0.0001	0.32
Full-time physicians in hospital (per 100 beds)	0.086	0.041	0.36	0.0417	0.07
Age 65+ workers (%)	0.045	0.015	0.49	0.0036	0.16
Self-reported health status: good (%)	0.041	0.018	0.34	0.0295	0.08

Public health nurses (per 100,000 population)*	0.034	0.008	1.08	< 0.0001	0.30
Full-time physicians in hospital (per 100 beds)*	0.084	0.049	0.35	0.0941	0.04

Women					
Public health nurses (per 100,000 population)	0.039	0.009	1.06	0.0001	0.28
Age 65+ workers (%)	0.041	0.018	0.39	0.0235	0.09
Self-reported health status: good (%)	0.045	0.021	0.32	0.0419	0.06

Public health nurses (per 100,000 population)*	0.038	0.006	1.04	0.0002	0.26

These models were adjusted for the proportion of people aged 65+ and population density (log-transformed ).

* : Adjusted for the proportion of people aged 65+, population density (log-transformed ), per capita income, and ordinary income/expenditure balance ratio

*β*: Partial regression coefficient

SE: standard error

## DISCUSSION

The use of ecological observations based on nearly 200 broad-ranging variables obtained from national surveys without any missing data yielded interesting results regarding factors related to Japanese health expectancy. Our study design permits us to propose the following hypothesis: As revealed by multivariate regression analysis, the large number of PHNs per unit population, a high proportion of older workers, and a high proportion of good self-reported health status potentially explain the regional differences in DFLE65s for both genders.

The results showed that after adjusting for confounding factors, only the human resource factors of the number of PHNs and physicians were associated with DFLE65s; however, medical infrastructure such as hospitals, clinics, and beds were not significantly associated with them. Particularly, the number of PHNs contributed the most to the DFLE65 differences. The ecological study in Spain showed that the large number of physicians was significantly correlated with long DFLE65,^18^ but it was insignificant in multivariate analysis. Further, the number of beds and the average length of stay in hospitals were not correlated with DFLE65s. The present study is in good agreement with this data. Therefore, medical human resources may be more important than medical infrastructure.

Meanwhile, the wealth of prefectures may confound the association. In other words, richer prefectures may employ of these professionals. In fact, per capita income and ordinary I/E balance ratio were correlated with the number of these professionals. The per capita income may indicate the wealth of a prefecture, and the ordinary I/E balance ratio is an indicator of the flexibility of the economy. In other words, if the I/E ratio is lower, the prefecture can use its operating expenses (such as employment cost or debt expenditure) in a more flexible manner.^48^ Indeed economic status may confound the number of PHNs, but the multivariate models that included the economic indicators showed that the number of PHNs per unit population was associated with DFLE65s independent of any economic condition.

With regard to the direction of the causality between the number of PHNs and DFLE65s, as the main practitioners of public health, PHNs in greater numbers can enhance the capacity of community health services.^49^ Ojima indicated that the number of PHNs per 10,000 population in Japanese municipalities was positively correlated with obtaining effective success of health care programs.^50^ Therefore, their activities may indirectly contribute to prolonging DFLE65s. Conversely, even if we assume that the longer DFLE65s result in higher numbers of PHNs in a prefecture, it is difficult to hypothesize a mechanism for the causal pathway. Thus, we hypothesized that medical human resources, particularly PHNs, were more important than other medical environment variables in prolonging the health expectancy of older people in Japan.

Although studies from the United Kingdom^17^ and Spain^18^ found an inverse association between unemployment rate and DFLE at birth, our multivariate analyses showed that neither unemployment rate nor proportion of workers were associated with DFLE65s. However, the large proportion of older workers was significantly associated with long DFLE65s. Unemployment rate and proportion of workers are indicators for all adult age groups, while DFLE65 is an indicator for the older population. Thus, the gap may explain the insignificant associations. Our study revealed the association between the employment condition of the older people and DFLE65s, which was indicated by the European studies as the association between unemployment rate and DFLE at birth. Thus, although the observed age groups were different, the result of our study was in good agreement with the European studies.

The education level is known as an individual^51^^-^^56^ and regional (contextual)^57^^,^^58^ determinant of health. In terms of its association with health expectancies, studies in Finland,^6^ Spain,^18^ and North America^19^^,^^20^ reported that low education level or literacy rate was associated with short health expectancies. The results of present study did not agree well with these studies. The differences in educational conditions may partly explain this discrepancy. For example, illiteracy rate was 0.2 percent in Japan in 1990 (the year of the last literacy survey)^59^ and 4.0 percent (range: 0.60-10.5) in Spain (1986).^18^ Thus, the Japanese regional differences in illiteracy rates might be too small to be associated with DFLE differences. Some cohort studies support our study. A comparative study of middle-aged people from Japan and England revealed that a higher education level was significantly associated with better behavioral and biological health indicators in England;^60^ however, it was not statistically significant in the case of Japan. Anzai et al. revealed a weaker relationship between education level and health practices parameters in Japan as compared to that in Europe and the United States.^61^ Although DFLE was not directly analyzed in these studies, the abovementioned factors are known to be the risk factors for physical disability in old age.^62^^,^^63^

The fact that the DFLE of older people has ecological-level associations with employment, but is not associated with education level, suggests that in Japan, employment of older people is a more important factor for prolonging the DFLE as compared to education level. However, the association between SES and Japanese DFLE is still unclear and warrants examination.

Self-reported health status is key to understanding the effects of other psychosocial influences on health. The result is consistent with a number of papers investigating self-reported health,^64^^-^^66^ suggesting that the Japanese DFLE65 estimated by the unit of prefecture appropriately reflects its health status and that the DFLE65 can be practically applied to monitor the health of population.

DFLE has been introduced as an appropriate indicator for deciding the allocation of social and health resources.^17^^,^^67^ As shown by the descriptive statistics, regional differences in these explanatory variables were not insignificant. For example, the number of PHNs per 100,000 population exhibits a 3.6-fold disparity among the 47 prefectures. The current result shows the effects of such disparities on the difference in the DFLE of older people. Therefore, this study may contribute to redistribution of the resources in a regional public health policy.

The present study has some limitations. First, the study design is affected by the problem of ecological fallacy. For example, the association of the number of PHNs for DFLE65s may reflect the effects of other components of medical systems on prefectures. To avoid fallacious observation, we removed the variables that seemed insignificantly associated with DFLE65 while gathering data for variables, and the consistencies of the results with other studies were discussed after the analysis. The second problem is related to the data sources used. There is a temporal gap in the data sources. Hence, the results should be viewed with caution, particularly when the time relationship is considered. In addition, because all the data used were cross-sectional, the result was potentially influenced by the period effect for each period in which every survey was conducted. Third, there may be some factors that should have been measured using smaller units, such as municipalities, because the local characteristics of some factors could vary within the prefectural level. In this situation, data at the prefecture level are equalized, and their association with DFLE65s may be underestimated. However, for smaller units, the trade-off between unit size and precision of data presents yet another problem. Fourth, some independent variables were obtained from the data that included non-aged population. The difference in population may be important while putting forth a hypothesis of individual level association. However, this does not pose a problem when the effects of regional characteristics for individual or regional health are hypothesized. Finally, the DFLE65 calculated using cross-sectional data is less precise as compared to other health expectancies based on the longitudinal data of disability incidence.^8^^-^^10^ The DFLE could potentially be affected by the nondifferential misclassification of disability that may lead to underestimation of DFLE differences, and in turn, the associations between DFLE and explanatory variables. However, we cannot obtain Japanese health expectancies based on the longitudinal data, and to conduct such a longitudinal study does not conform with the study. With regard to the consistency of estimation, Miyashita et al. mentioned that the percentage of disabled population that was estimated for their DFLE calculation was similar to the results of other studies in 1990,^68^ although the methods were not exactly the same.^15^ Because the purpose of this study was to generate hypotheses regarding the factors that determine the differences in health expectancy in Japan as a preliminary step for further research, these limitations are insignificant.

In conclusion, the number of PHNs per 100,000 population, occupational status of older people, and self-reported health status are the factors that potentially explain the regional differences in the DFLE65s for both men and women in Japan. Particularly, the new finding that shows a positive and strong association between the large number of PHNs per 100,000 population and long DFLE65s warrants further study. The results were consistent with some other studies, which support the validity of the use of DFLE for health policy in prefectures. In the future, individual-level or multi-level analytic research should be conducted to confirm the existence of causal associations and regional contextual factors affecting the individual’s self-sufficient period, as well as to provide evidence for setting regional health policies.
